# A Central Role for JNK/AP-1 Pathway in the Pro-Oxidant Effect of Pyrrolidine Dithiocarbamate through Superoxide Dismutase 1 Gene Repression and Reactive Oxygen Species Generation in Hematopoietic Human Cancer Cell Line U937

**DOI:** 10.1371/journal.pone.0127571

**Published:** 2015-05-21

**Authors:** Humberto Riera, Valéry Afonso, Pascal Collin, Abderrahim Lomri

**Affiliations:** 1 Unidad de Reumatología, Nivel plaza del Instituto Autónomo Hospital Universitario de Los Andes. Mérida, Venezuela; 2 INSERM U1029, Laboratoire de l'Angiogenèse et du Microenvironnement des Cancers, Pessac, France; 3 UMR 8601, Laboratoire de Chimie & Biochimie Pharmacologique, Paris, France; Institute of Hepatology - Birkbeck, University of London, UNITED KINGDOM

## Abstract

Pyrrolidine dithiocarbamate (PDTC) known as antioxidant and specific inhibitor of NF-κB was also described as pro-oxidant by inducing cell death and reactive oxygen species (ROS) accumulation in cancer. However, the mechanism by which PDTC indices its pro-oxidant effect is unknown. Therefore, we aimed to evaluate the effect of PDTC on the human Cu/Zn superoxide dismutase 1 (SOD1) gene transcription in hematopoietic human cancer cell line U937. We herein show for the first time that PDTC decreases SOD1 transcripts, protein and promoter activity. Furthermore, SOD1 repression by PDTC was associated with an increase in oxidative stress as evidenced by ROS production. Electrophoretic mobility-shift assays (EMSA) show that PDTC increased binding of activating protein-1 (AP-1) in dose dependent-manner suggesting that the MAPkinase up-stream of AP-1 is involved. Ectopic NF-κB p65 subunit overexpression had no effect on SOD1 transcription. In contrast, in the presence of JNK inhibitor (SP600125), p65 induced a marked increase of SOD1 promoter, suggesting that JNK pathway is up-stream of NF-κB signaling and controls negatively its activity. Indeed, using JNK deficient cells, PDTC effect was not observed nether on SOD1 transcription or enzymatic activity, nor on ROS production. Finally, PDTC represses SOD1 in U937 cells through JNK/c-Jun phosphorylation. Taken together, these results suggest that PDTC acts as pro-oxidant compound in JNK/AP-1 dependent-manner by repressing the superoxide dismutase 1 gene leading to intracellular ROS accumulation.

## Introduction

Increases in reactive oxygen species (ROS) production and defects in the ROS-removing enzymatic system can cause serious damage to cells [1]. The necessity of maintaining the redox balance is underscored by the evidence that many apoptotic stimuli induce oxidative stresses directly or indirectly [1, 2]

Superoxide dismutases (SODs) constitute a family of antioxidant enzymes that catalyzes the conversion of superoxide anions to oxygen and hydrogen peroxide. Altered expression and mutations of SOD1 are implicated in a variety of neuropathological conditions such as familial amyotrophic lateral sclerosis and Down’s syndrome [3, 4]. Additionally, over-expression of Cu/Zn-SOD in mice blunted ethanol-induced activation of redox-sensitive transcription factor AP-1 and production of TNF-α and interleukin-6 [5].

In vitro, SOD expression suppressed JNK (also called stress-activated protein kinase, SAPK) and p38 phosphorylation and attenuated intracellular superoxide anion production and NADPH oxidase activity in TNF-α-treated cells [6]. JNK family members belong to the mitogen-activated protein kinase (MAPK) super family including extracellular signal-regulated kinases (ERKs) and the p38-MAPK family [7, 8]. The JNK pathway responds to diverse stimuli including mitogens, pro-inflammatory cytokines and environmental stresses [7–9]. Activation of JNK signal pathway by IL-1β and TNF-α in inflammatory conditions has been shown to require ROS as a signaling intermediate [10].

In vivo, over-expression of SOD1 by delivery of the SOD1 gene with an adenovirus (Ad.SOD1) decreases organ injury and increases survival in a rat model of liver transplantation through inhibition of JNK and TNF-α activities [11–13].

Pyrrolidine dithiocarbamate (PDTC) is a synthetic low-molecular-weight thiol compound that has been initially described as an antioxidant agent [14, 15]. However, due to its ionophore properties [16, 17] or the possibility to be enzymatically converted into the reactive intermediate sulfenic acid [18], PDTC may also act as a pro-oxidant agent [17]. Several investigators have shown that PDTC inhibits the expressions of pro-inflammatory genes in response to inflammatory mediators such as TNF-α and LPS in vivo [19, 20] and in vitro [21, 22] via suppressing NF-κB activation. NF-κB is a transcription factor involved in the expression of a wide range of genes, most of which code for proteins that play a role in immunity and inflammation. In vivo, oral administration of PDTC inhibits tumor growth, migration and angiogenesis of breast cancer via inhibiting autocrine and paracrine effects of VEGF through the reduction of NF-κB activation and VEGF expression in female mice [23]. In contrast, others have reported that PDTC activates NF-κB, which seems to be depended on its dose and the presence of metal ions in cells [24]. Furthermore, Meisner et al. [25] showed that plasma concentration of TNF-α was slightly augmented in PDTC-treated animals.

It has been reported that the antioxidant activity of PDTC induces apoptosis in some tumor cell lines, such as colorectal [15] and prostatic carcinoma cells [26], but may also lead to a reduction of cell growth inhibition of colorectal cancer cells by photosensitization [27]. The pro-oxidant activity of PDTC has been described to induce cell death in human acute myelogenous leukemic cells [28], but also cell proliferation in a murine thymoma cell model [29]. Therefore, PDTC may be considered as a functionally versatile molecule, which acts in a context specific manner depending on the specific cellular model and microenvironment. We have recently reported that TNF-α inhibits SOD1 transcription in U937 cells through JNK/AP-1 pathways. However, PDTC or N-acetysysteine (NAC) treatments were unable to block TNF effects [30].

In this study, to elucidate the abilities of PDTC-induced oxidative stress combined with ROS production, we investigated the effect of PDTC on the antioxidant gene SOD1, JNK activation and NF-κB inhibition in hematopoietic human cancer cells.

We found that PDTC decreases SOD1 transcripts, protein and promoter activity. SOD1 repression by PDTC was associated with an increase in oxidative stress. PDTC increased binding of activating protein-1 (AP-1) in dose dependent-manner suggesting that the MAPkinase up-stream of AP-1 is involved. Ectopic NF-κB p65 subunit overexpression induced a marked increase of SOD1 transcription in the absence of JNK activity. Finally, PDTC represses SOD1 in U937 cells through JNK/c-Jun phosphorylation. Taken together, these results suggest that PDTC acts as pro-oxidant compound in JNK/AP-1 dependent-manner by repressing the superoxide dismutase 1 gene leading to intracellular ROS accumulation. Our results suggest that inhibition of SOD1 by PTDC has potential clinical application as a single agent, or in combination with other known cancer therapeutics.

## Materials and Methods

### Cell culture and agents

U937 human myeloid leukemia cells (American Type Culture Collection, Rockville, MD) were grown in RPMI 1640 medium supplemented with 5% heat-inactivated FCS, 200 mM glutamine, 100 units/ml penicillin, and 100 μg/ml streptomycin. Immortalized fibroblast cell line derived from JNK_1_^–/–^-JNK_2_^–/–^ mouse embryos [31], in which targeted disruption of JNK1 and JNK2 has been performed simultaneously was a kindly provided by Dr. A. Mauviel [32]. These cells were grown in RPMI 1640 medium supplemented with 10% heat-inactivated fetal calf serum, 2 mM glutamine and antibiotics (100 U/ml penicillin, 50 μg/ml streptomycin-G and 0.25 μg/ml Fungizone). A protease inhibitor cocktail, the JNK inhibitor SP600125 and PDTC were purchased from Calbiochem (San Diego, CA). The anti-phospho-c-Jun (KM-1), anti-c-Jun (H-79) and anti-JNK1 (C-17) antibodies were obtained from Santa Cruz Biotechnology Inc. (Heidelberg, Germany). Anti-phospho-SAPK/JNK (Thr183/Tyr185) was purchased from Cell Signaling Technology (New England Biolabs, Saint Quentin Yvelines, France). Miscellaneous reagents such as chemicals and salts were from Sigma-Aldrich Chemicals. All reagents were of the highest grade commercially available. In experiments involving inhibitors, cells were pre-incubated with the inhibitor 1 h before the treatment. 2′, 7′-dichlorofluorescein diacetate was from Molecular Probes (Cergy Pontoise Cedex, France).

### Plasmids

SOD1 full-length (1.5 kb) reporter plasmid containing the firefly luciferase gene and its various 5′-flanking regions were kindly provided by Dr. C. Jaulin [33].

### Transient transfection and luciferase assays

The activities of full-length or truncated SOD1 promoter constructs were analyzed in U937 cells. Briefly, cells were collected by centrifugation, resuspended in serum-free RPMI media containing 250 mM sucrose, transferred to a cuvette in the presence of different SOD1 constructs or promoterless pGL2-Basic control vector plasmid, and then electroporated using a Bio-Rad gene pulser as previously described [34]. Following electroporation, cells were resuspended in media containing 5% FCS and plated in 12-well tissue culture dishes. Cells were treated following transfection with PDTC for 24 h and then collected and lysed in luciferase lysis buffer (Promega). Luciferase assay was performed according to the manufacture's instructions using a Monolight luminometer (Analytical Luminescence Laboratory, Sparks, MD).

### Preparation of nuclear extracts and EMSA

Nuclear extracts from 3 × 10^6^ cells stimulated by PDTC at various doses were prepared according to the protocol of Dignam et al. [35]. They were rapidly frozen in liquid nitrogen and stored at −80°C until used. Protein concentration was measured with the BCA protein assay kit (Pierce Biotechnology, Rockford, IL) with bovine serum albumin (BSA) as standard. Double-stranded AP-1 oligonucleotide used for EMSA was from Promega. 5′-End labeling of double-stranded oligonucleotide was performed by polynucleotide kinase reaction (Promega) using 3.5 pmol of oligonucleotide and 25 μCi of [γ-^32^P]ATP (3000 Ci/mmol at 10 mCi/ml; Perkin-Elmer). Labeled probe was purified by spin column chromatography (G-50, Amersham Pharmacia Biotech). Nuclear extracts (1–5 μg) were incubated with radiolabeled AP-1 probe (2 × 10^4^ cpm) in binding buffer (Promega) for 20 min at room temperature. In competition studies, samples were incubated with a 100× excess of unlabeled AP-1 oligonucleotide or AP-1 antibody (2 μg) 10 min prior to probe addition. Anti-AP-1 antibody was purchased from Santa Cruz Biotechnology. Resulting complexes were electrophoresed through 4% nondenaturing acrylamide gels in 0.5× Tris borate-EDTA running buffer (200 V, room temperature). The gel was analyzed with a Storm 860 Molecular Imager (GE HealthCare, Piscataway, NJ).

### Western blot analysis

U937 cells were first serum-starved for 24 h, treated with or without PDTC at the indicated concentration and time, rinsed twice with PBS, lysed in luciferase lysis buffer (Promega), and then centrifuged at 12,000 g for 5 min at 4°C to remove cellular debris. Ten micrograms of proteins per lane was analyzed by electrophoresis through 12% SDS/PAGE in a Bio-Rad minigel system (100 V, 80 min) and then electrophoretically transferred to Amersham polyvinylidene difluoride-Hybond-P membrane (300 mA, 80 min). Blots were saturated overnight with 1% blocking solution (Roche Diagnostics France) in Tris-buffered saline solution (50 mmol/L Tris-HCl, 150 mmol/L NaCl) containing 0.1% Tween 20 and incubated with anti-human Cu/ Zn-SOD1 (1:5000; Rockland Immunochemicals Inc., Gilbertsville, PA), anti-phospho-JNK (1:1000), anti-JNK (1:1000) (Cell Signaling Technology, Beverly, MA), anti-c-Jun (1:500; Santa Cruz, CA), or anti-actin primary (1:500) antibodies for 1 h at 37°C. For experiments with the JNK inhibitor SP600125, cells were pre-incubated 1 h prior to PDTC treatment. After incubation with primary antibodies, membranes were washed, incubated with horseradish secondary antibody (1:15,000) for 30 min, and washed again. The antibody complex was detected with ECL Reagent Plus (Amersham), and bands were visualized with a Storm 860 Molecular Imager. Bands were quantitatively analyzed, and results expressed as ratio of control after normalization to β-actin.

### SOD1 activity assay

To evaluate the effect of PDTC on SOD1 activity, U937 cells were serum starved over night prior treatment with 1 μM PDTC for 24 h. At the end of treatment, cell pellet was then homogenized in cold 20 mM HEPES buffer, pH 7.2, containing 1 mM EGTA, 210 mM mannitol and 70 mM sucrose. Quadruplicate 10 μl aliquots were assayed for SOD activity by spectrophotometer using an SOD Assay kit (Cayman Chemical Co, Ann Arbor, MI). SOD2 activity was determined in the presence of 5 mM NaCN. SOD1 activity was estimated by subtracting the SOD2 activity from the total activity. Protein concentrations in the homogenates were measured with a Coomassie Plus Protein Assay Reagent (Pierce Chemical, Rockford, IL). Data are presented as means ± SD, and the significant variation was determined using a two-tailed *t* test.

### Measurement of reactive oxygen species

ROS generation was assessed using the probe 2, 7-dichlorofluorescein (DCF). Briefly, cells were serum starved for 24 h and then washed with PBS to eliminate the media with phenol red. The membrane-permeable diacetate form of the dye (reduced DCF (DCFH-diacetate)) was added to the cells (10^6^ cells/ml) at a final concentration of 1 μM in PBS for 10 min at 37°C. Within the cell, esterases cleave the acetate groups on DCFH-diacetate, thus trapping the reduced probe (DCFH) intracellularly. ROS in the cells oxidize DCFH, yielding the fluorescent product DCF. At the end of incubation, cells were washed with PBS before treatment with PDTC for 30 min at 37°C. At the end of treatment, cell pellets were washed with PBS and then cells lysed in deionized water. Cells were handled in the dark throughout the experiment from cell culture to the spectrophotometer measurements. The fluorescence intensities were measured with a Hitachi spectrofluorimeter, with an excitation wavelength of 485 nm and emission at 530 nm (Hitachi, Ltd, Tokyo, Japan). The change in fluorescent intensity for each experimental group was first normalized with the amount of proteins per culture and then expressed as a percentage of respective control values, and then values for each treatment were compared to the control values.

### Quantitative real-time PCR

Total RNA from control and PDTC treated cells was subjected to cDNA synthesis using the Superscript first strand cDNA synthesis system (Invitrogen). The relative quantification of specific mRNAs was performed by real-time PCR using the StepOnePlus. Real-Time PCR System and Power SYBR Green PCR Master Mix (Applied Biosystems) according to the manufacturer’s instructions. In brief, the mixture of the reaction consists in 20 μl total volume of 2 μl of cDNA, 2 x QuantiTect SYBER Green PCR Master Mix, and 0.5 μM of the forward and reverse primers obtained from Primer Bank (http://pga.mgh.harvard.edu/primerbank/index.html). Real-time PCR analysis was performed in duplicate using iQSYBR Green Mix with an iCycler thermal cycler. The data were analyzed using comparative threshold cycle method using GAPDH as internal control.

### Image analysis and statistical analysis

Image analysis was performed using ImageJ, a public domain Java-based image-processing program inspired by NIH Image. Results are expressed as mean ± standard deviation. Comparisons between groups were made with the Student’s t-test. Differences were considered significant when P<0.05.

## Results

### PDTC inhibits Cu/Zn-SOD1 mRNA transcripts and protein

To exclude an ability of PDTC to induce cell death, we first investigated the effect of PDTC on cell viability using MTT assay. Loss of viability was not observed following PDTC exposure at various doses (0.5–5 μM) after 24 h (data not shown).

**Fig 1:**
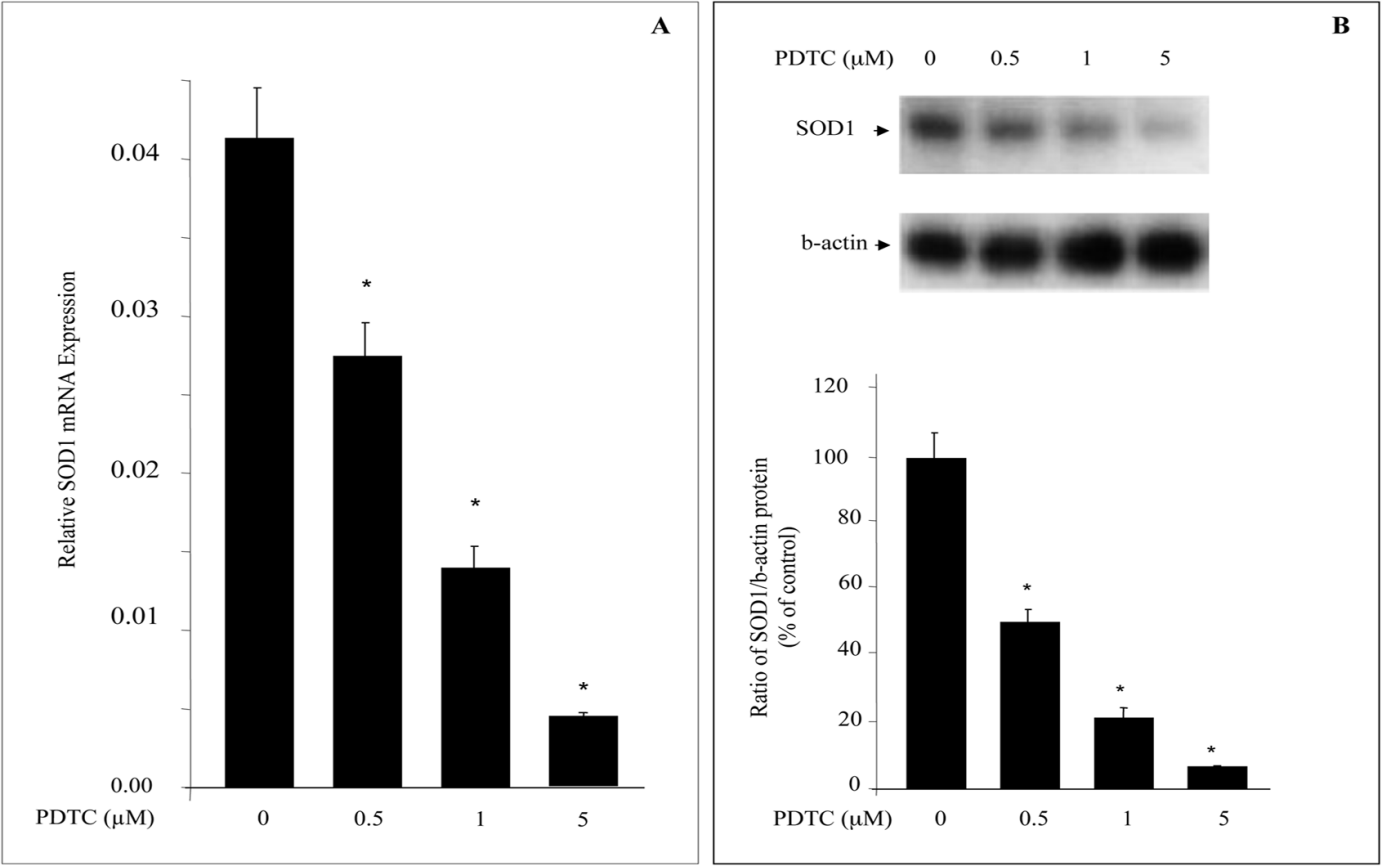
PDTC inhibits endogenous SOD1 mRNA and gene product. Serum-starved U 937 cells were treated with various doses of PDTC for 24 h. A; Quantitative real time PCR was performed for SOD1 mRNA as described in material and methods, and data were normalized to GAPDH mRNA. Each data point is the average of triplicate determinations ± SD (*, P < 0.05, in comparison to untreated cells). B; Protein levels of SOD1 were measured by Western blotting, and normalized to β-actin. SOD1 protein was reduced in PDTC treated cells. Computer-assisted densitometry confirmed the significant decrease of SOD1 expression in the presence of PDTC. The intensity of the band was normalized to that of β-actin. *P < 0.05.

We next investigated whether PDTC-induced ROS generation could be linked to a decrease in the anti-oxidant gene SOD1. U937 human myeloid leukemia cells were treated with different doses of PDTC and SOD1 mRNA levels were determined by real- time PCR analysis. As shown in Fig 1A, the U937 cells express constitutively high levels of SOD1 mRNA. After 24 h of PDTC treatment, human SOD1 transcript levels decreased significantly. The decline in SOD1 mRNA was observed (40%) at the lowest PDTC dose (0.5 μM) and maximal inhibition (80%) occurred at 5 μM PDTC (Fig 1A). SOD1 mRNA inhibition by PDTC was associated with a marked reduction of SOD1 protein at different doses after 24 h of treatment (Fig 1B). We chose to use a 24 h treatment to measure SOD1 mRNA to relate these results to the transfection studies with the SOD1 reporter constructs.

### PDTC down-regulates Cu/Zn-SOD1 promoter activity

**Fig 2:**
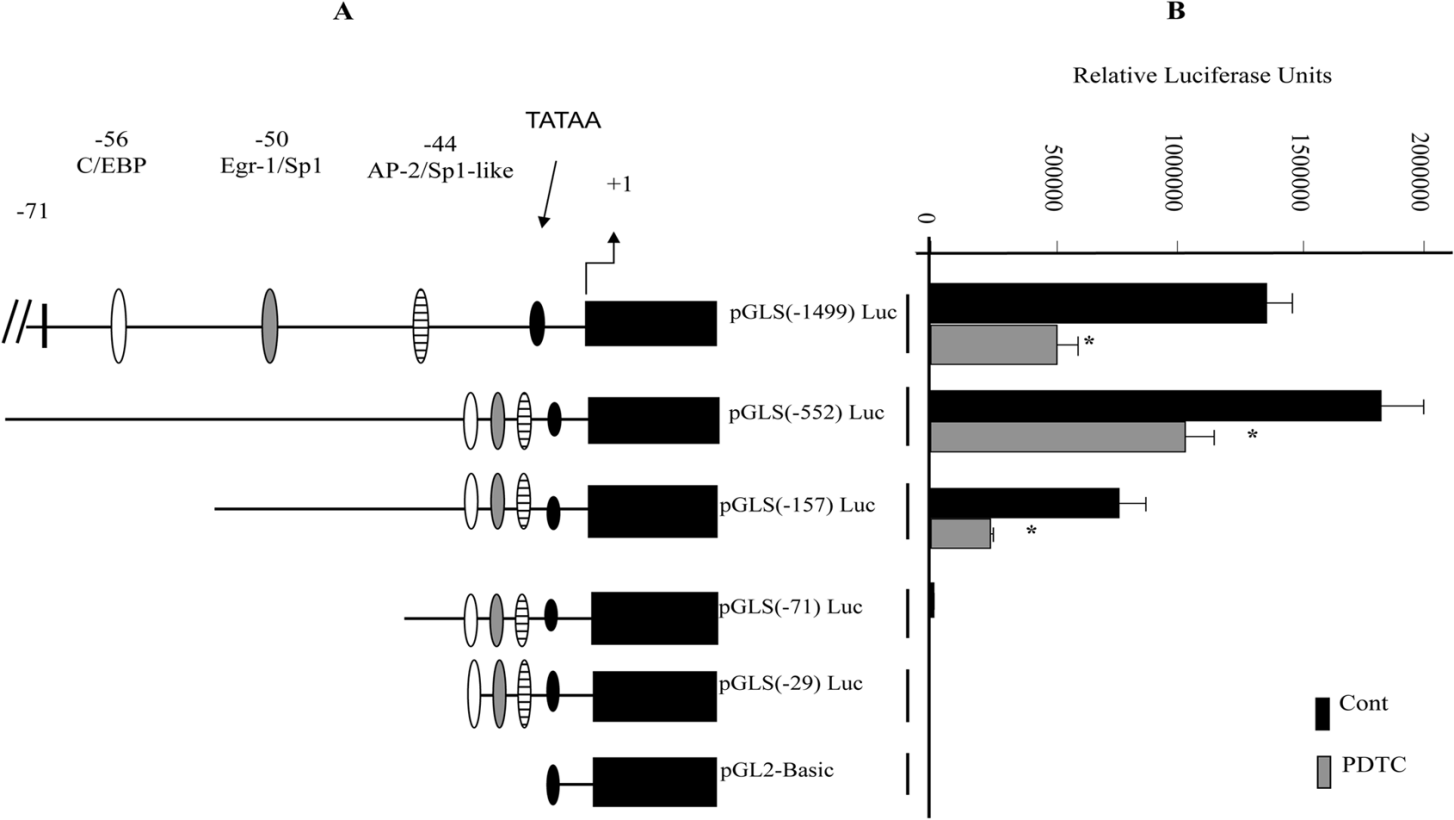
PDTC repression of the SOD1 promoter from a 5′ deletion series linked to the luciferase gene in U937 cells. A; The 5′ boundaries of plasmids containing various truncations of the SOD1 promoter are shown with the C/EBP, Egr-1/Sp1 sites indicated. B; SOD1 constructs (1 μg) or promoter-less pGL2-Basic control vectors were transiently transfected into U937 cells as described under Experimental procedures. The relative luciferase activity expressed by each plasmid in the absence or presence of 1 μM PDTC for 24 h is shown with SD. Statistical analysis was performed by using Student's t test (*P < 0.01) by comparing values from PDTC-treated cells with control values for each SOD deletion. Results are representative of four independent experiments.

In order to confirm the real PCR data and to localize the DNA element involved in the transcriptional repression of the SOD1 gene by PDTC, we used different SOD1 truncated reporter constructs linked to luciferase gene (Fig 2A) to transfect the U937 cells. As shown in Fig 2B, constructs pGLS-1499 to -157 bp displayed basal promoter activity, which was inhibited by PDTC. Removal of the -157 to -71 region reduced drastically promoter basal activity (Fig 2B). PDTC effect was abolished when the promoter was deleted to -29 bp. This region contains binding sites for multiple transcription factors, including C/EBP, E2F, and Sp1/Egr-1 as previously reported [30]. The luciferase expression level from cell transfected with deletion -29 was similar to that obtained with the promoterless pGL2-Basic control vector, which was barely detectable. These data were confirmed using the human U2OS osteosarcoma cell line (data not shown).

### Gel-shift analysis of the effects of PDTC on nuclear proteins bound to the AP-1 probe

**Fig 3:**
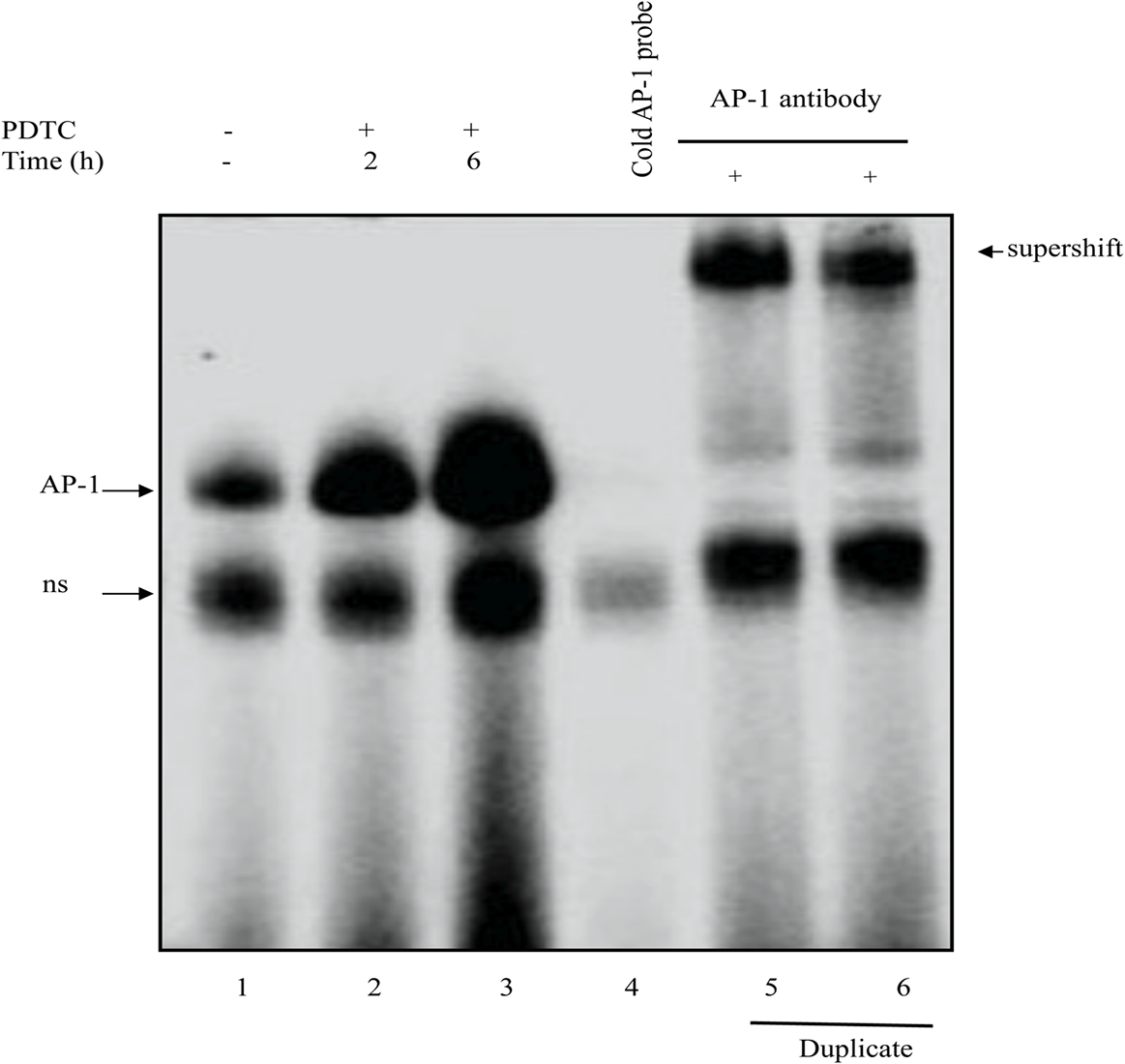
Gel-shift analysis of the effects of PDTC on nuclear factors bound to the AP-1 probe. EMSA experiments were performed using nuclear extracts from control (lane 1) and PDTC-treated U937 cells for 2 (lane 2) and 6 h (lane 3) and a ^32^P-labeled double-stranded AP-1 oligonucleotide as a probe. Competition experiments were performed by pre-incubating nuclear extracts from control cells with 100 x excess cold AP-1 double-stranded oligonucleotides (lane 4) or anti-AP-1 antibody (lane 5 & 6 are duplicates) for supershift prior incubation with labeled AP-1 probe addition. Results are representative of three independent experiments. Unspecific bands (ns)

SOD1 expression is regulated by many transcription factors that can act as activators or inhibitors of SOD1 transcription. In fact, SOD1 proximal promoter (−157) contains several motifs that are homologous to known *cis*-acting regulatory elements. In particular, we have reported [30] that Sp1 transcription factor is an activator of SOD1 transcription, whereas AP-1 is involved in SOD1 repression. In particular, AP-1 inhibits SOD1 transcription by sequestrating essential co-activators, rather than binding to the SOD1 gene promoter [33]. Therefore, we investigated the binding activity of AP-1 by EMSA in nuclear protein extracts. Incubation of nuclear extracts from PDTC treated cells (Fig 3, lanes 2 and 3) with consensus AP-1 oligonucleotide resulted in a significant increase of retarded DNA-protein complex with respect to control cells (Fig 3, lane 1). The specificity of the binding was demonstrated using the appropriate competition assay with excess of AP-1 unlabeled probe (Fig 3, lane 4). In addition, AP-1 complex was completely supershifted by anti-AP-1 antibody (Fig 3, lanes 5 and 6), confirming the specificity of the binding. This result suggests that AP-1 is involved in PDTC negative effect on SOD1 promoter activity by interfering with the binding of other factors as we have previously suggested [30].

### Activation of JNK pathway is necessary for the PDTC-induced SOD1 repression

**Fig 4:**
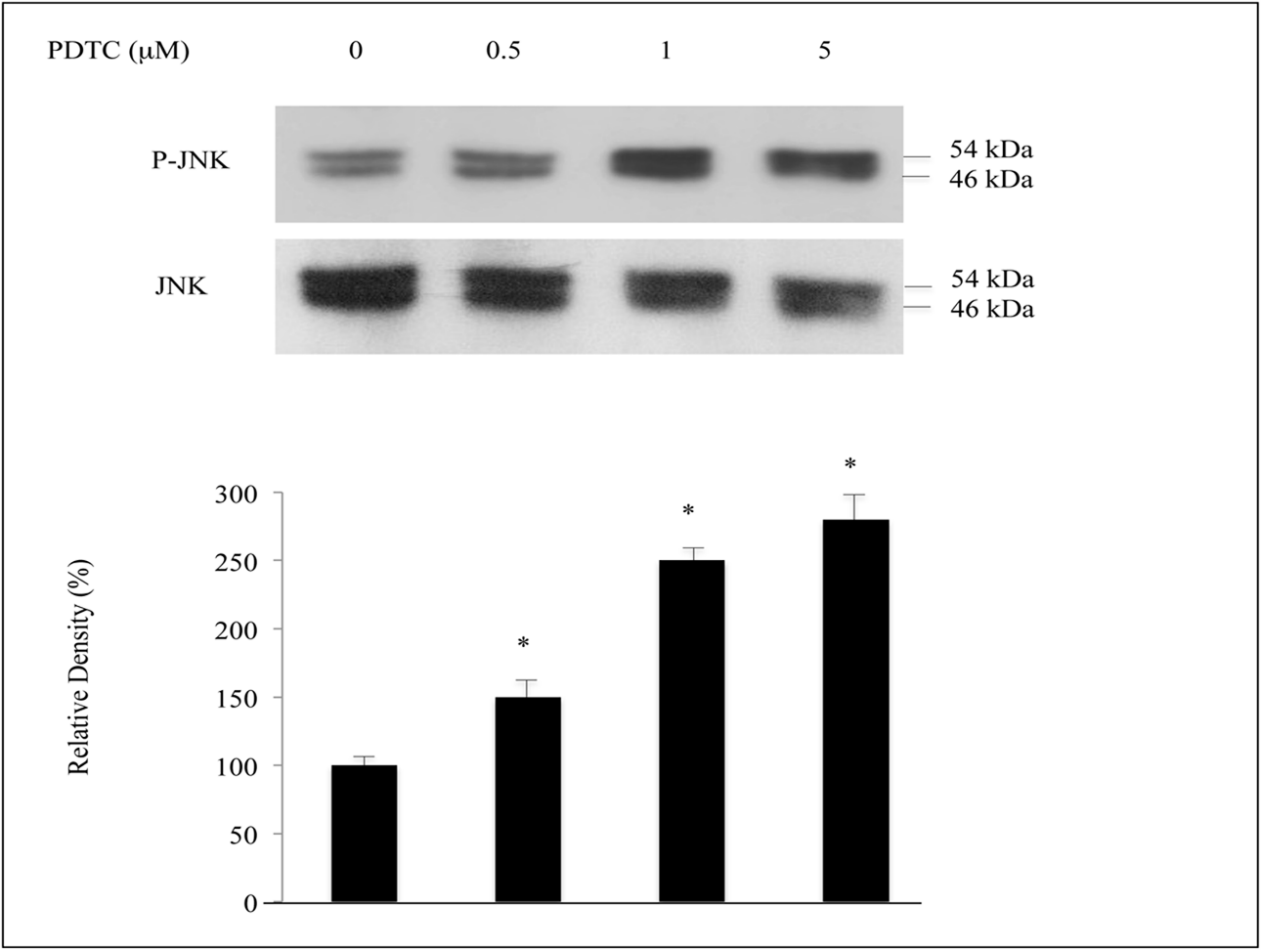
PDTC treatment promotes JNK activation in dose dependent-manner. Upper panel, whole-cell lysates were taken at the end of treatment after isolation and analyzed by Western blotting as indicated in material & methods. The blots are representative of three independent experiments. Lower panel, semiquantification of representative blots using densitometric analysis.

Since several distinct signal pathways can induce the activation of AP-1, we next determined the intracellular signals that regulate the AP-1 activation in U937 cells in response to PDTC. We investigated whether PDTC modulates the activity of the MAPK JNK intracellular pathway. Exposure of the U937 cells to PDTC resulted in phosphorylation/activation of JNK in dose-dependent manner. However, JNK protein remained unaltered after treatment with PDTC (Fig 4). These results suggest that the SAPK/JNK pathway contributes to AP-1 activation in the PDTC-stimulated U937 cells.

### Endogenous JNK pathway controls negatively NF-κB activity

**Fig 5:**
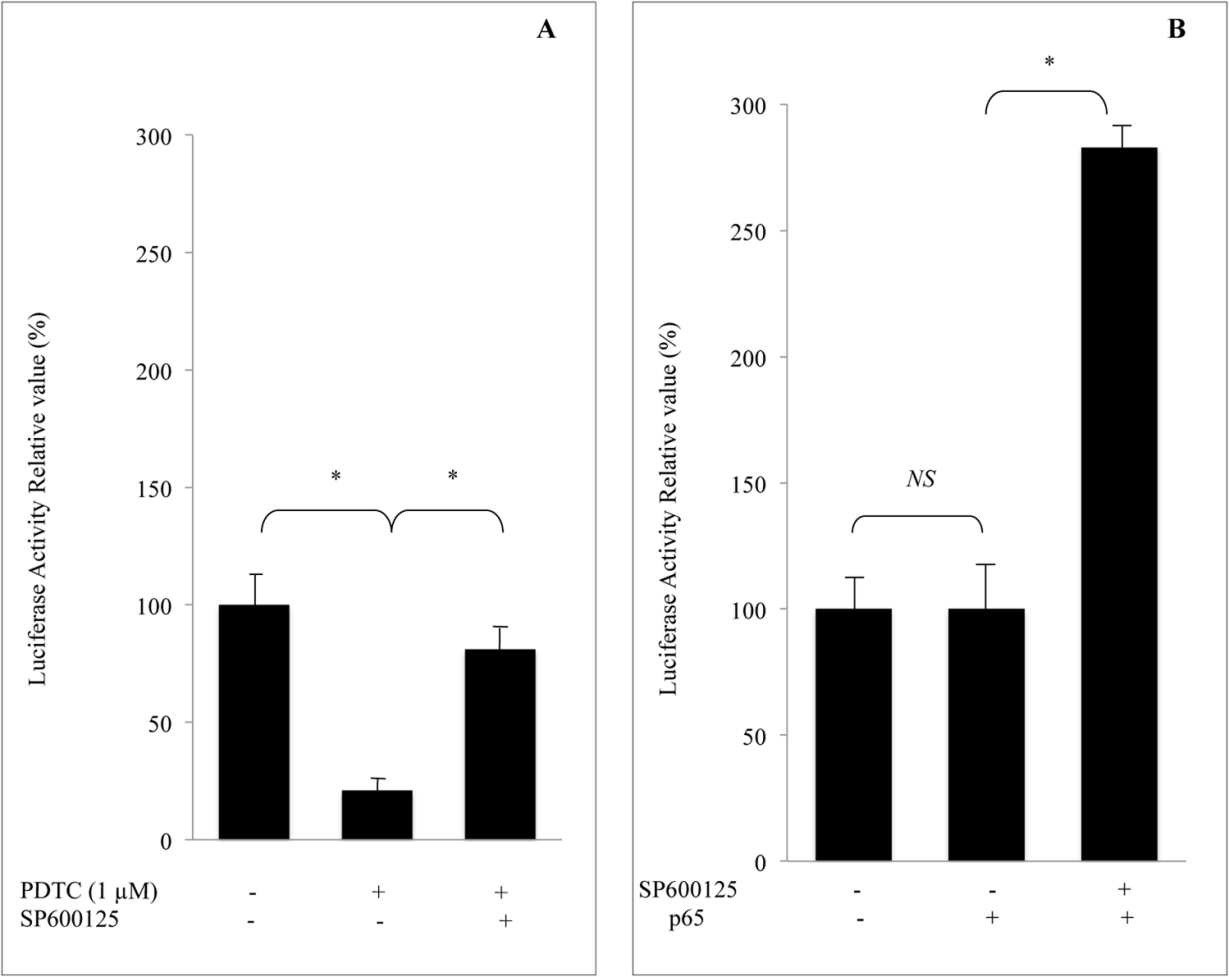
Endogenous JNK pathway controls negatively NF-κB activity. A; Repression of SOD1 transcription by PDTC is abolished by JNK inhibitor SP600125. Transfected U937 cells were pre-incubated with 10 μM SP600125 for 1 h, and then treated with 1 μM PDTC over-night prior cell lysates. B; Activation of SOD1 transcription by NF-κB sub-unit p65 is JNK-dependent. The results are presented as mean ± SE from 4 separate experiments. *P < 0.01 vs. control. NS, not significant.

To further explore the significance of the JNK pathway, we investigated the expression of the SOD1 gene in the presence of pathway-specific inhibitor. Transcription activity of SOD1 promoter in the presence of 1 μM of PDTC was examined after the cells were pre-incubated with the JNK inhibitor SP600125 (10 μM), As shown in Fig 5A, pre-incubation of the cells with JNK inhibitor prevented the repression of SOD1 transcription by PDTC. Furthermore, in order to evaluate the relationship between JNK pathway and NF-κB activity, we overexpressed the NF-κB p65 sub-unit in cells transfected with SOD1 promoter in the absence of PDTC. No change was observed in these conditions. In contrast, when JNK pathway was inhibited prior NF-κB p65 over expression, a marked increase in promoter activity was obtained (Fig 5B). These results suggested that NF-κB acts as transactivating factor of SOD1 gene and that a negative cross talk between JNK pathway and NF-κB activity exists in U937 cells.

### PDTC is inactive in JNK deficient cells

**Fig 6:**
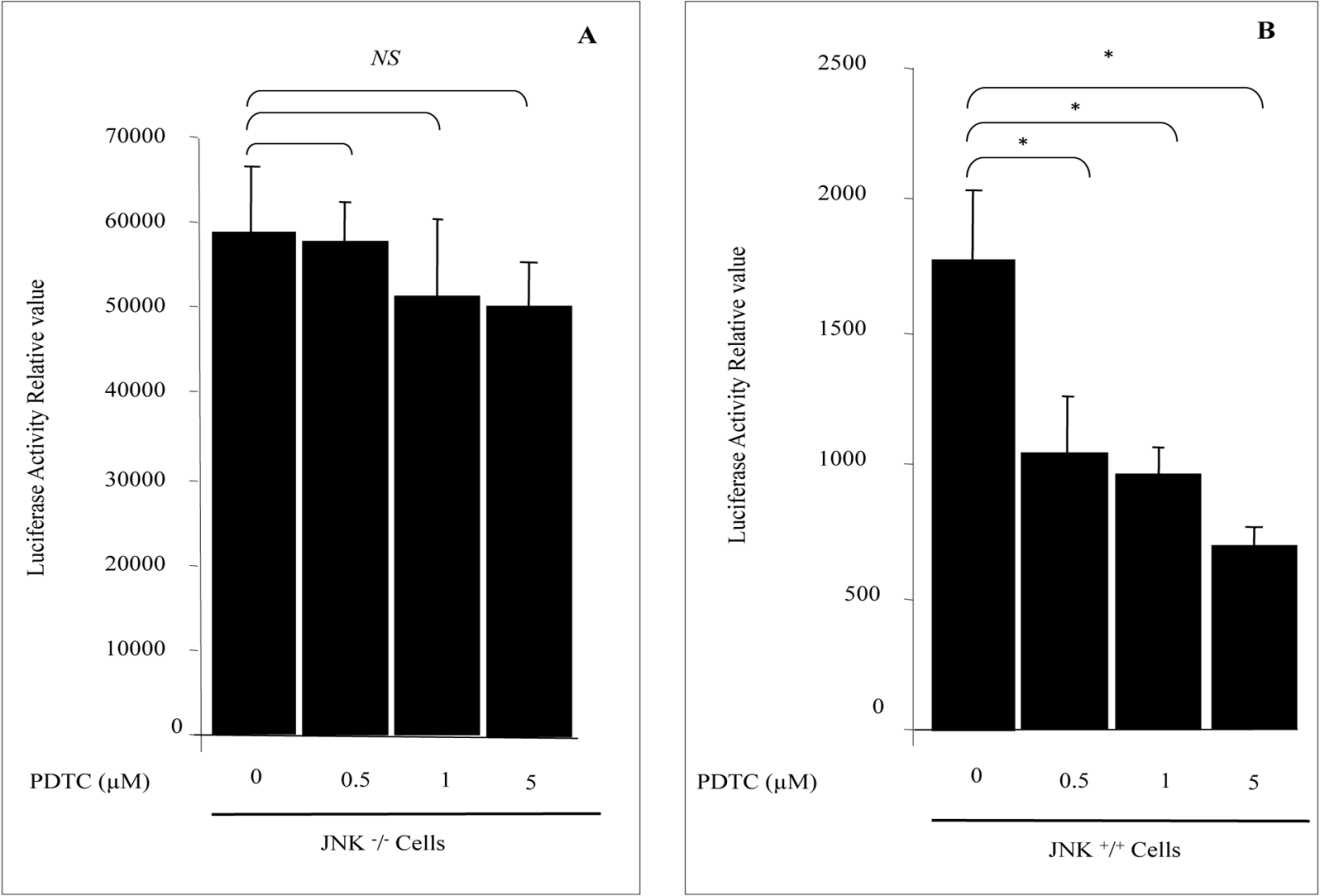
PDTC is inactive in JNK deficient cells. A; PDTC represses SOD1 activity in JNK ^+^/^+^ cells. B; SOD1 activity is not sensitive to PDTC in JNK deficient cells. Serum starved JNK ^-^/^-^ & JNK ^+^/^+^ cells were treated with various doses of PDTC in RPMI1640 media supplemented with 1% serum. After 24 h of treatment, transfected cells were lysed and SOD1 luciferase activity in cell lysates was assayed as described in material & methods. The results are presented as mean ± SE from 4 separate experiments. *P < 0.01 vs. control. NS, not significant.

Because the use of commercial inhibitors may have some times second effects on cell function, we decided to use JNK deficient cells to confirm our finding with SP600125 known as specific inhibitor of JNK pathway. SOD1 basal transcriptional activity in immortalized mouse fibroblast cell line *JNK1* ^*-/-*^ *–JNK2* ^*-/-*^ was increased by 33 fold (Fig 6A) compared to mouse fibroblast cell line wild type (Fig 6B). Treatment of JNK deficient cells with PDTC did not affect SOD1 promoter activity, in contrast to wild type cells (Fig 6). These data demonstrate that active intracellular JNK pathway is necessary to PDTC action on SOD1 transcription.

### JNK activation by PDTC promotes c-Jun phosphorylation

**Fig 7:**
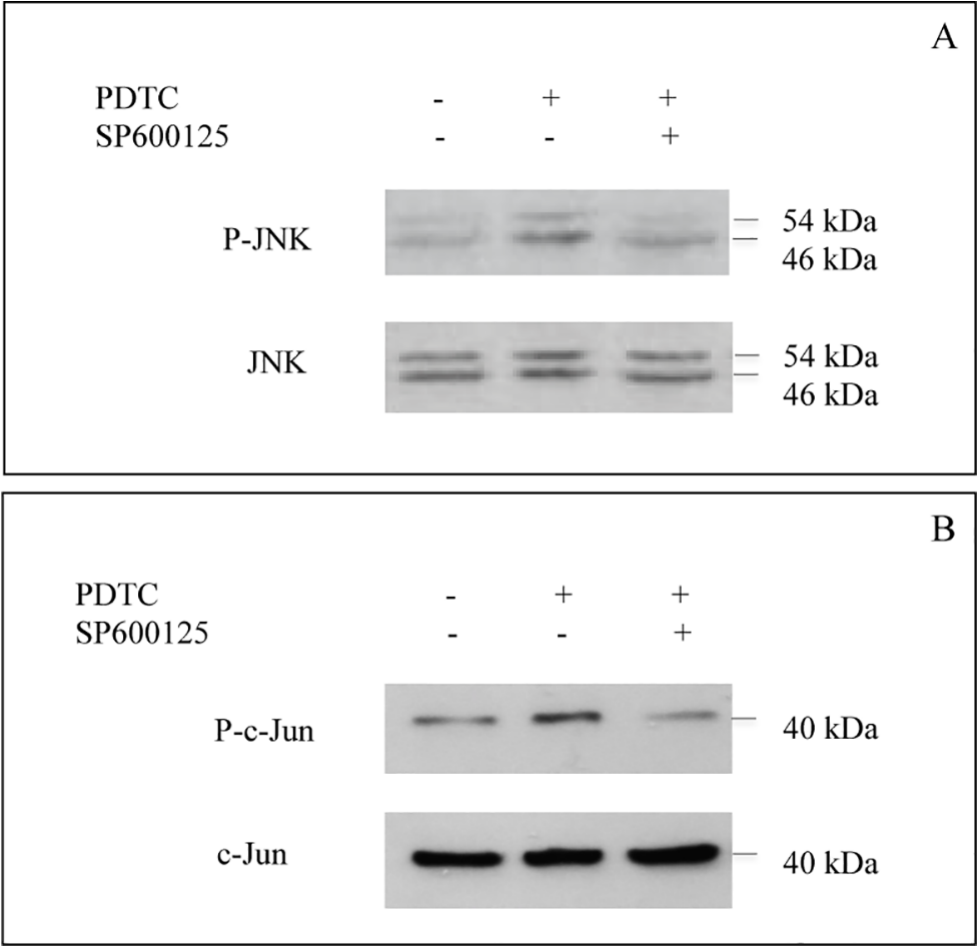
JNK activation by PDTC promotes c-Jun phosphorylation. A; Activation of JNK phosphorylation by PDTC is abolished by SP600125. U937 cells were pre-incubated with 10 μM SP600125 for 1 h, and then treated with 1 μM PDTC for 20 min prior cell lyses. B; PDTC-induced c-Jun activation is dependent on JNK phosphorylation in U937 cells. The data shown are means ± SD from 4 independent experiments.

As JNK signaling is known to regulate AP-1 activity, we examined the extent of phosphorylation of JNK substrate, c-Jun in the presence of PDTC in U937 cells. As shown in Fig 7, activation of JNK pathway up stream of c-Jun by PDTC (Fig 7A) resulted in phosphorylation of c-Jun protein (Fig 7B). However, both JNK and c-Jun total proteins remained unaltered after treatment with PDTC.

### PDTC inhibits SOD1 enzymatic activity in JNK +/+ cells

**Fig 8:**
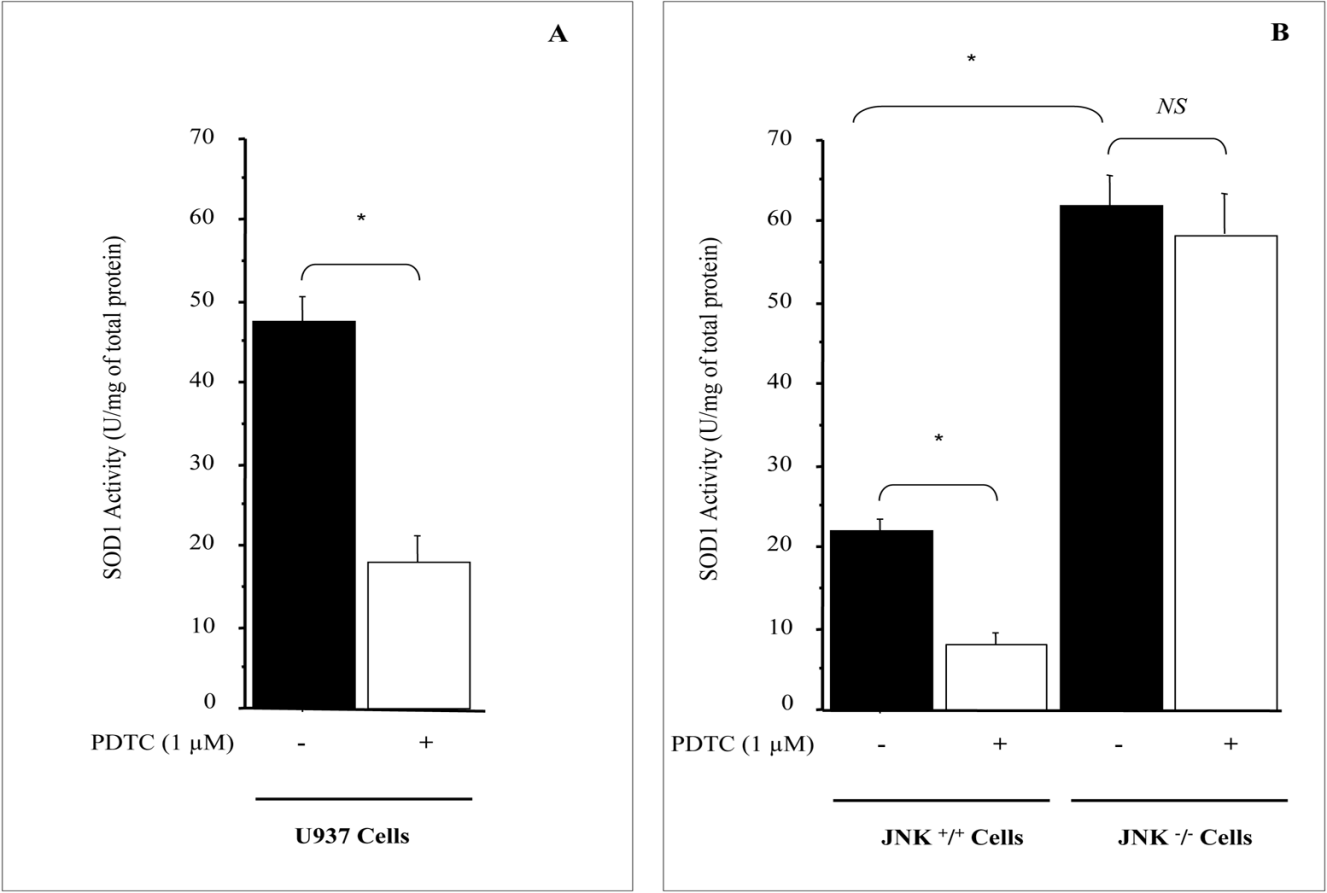
PDTC inhibits SOD1 activity in JNK ^+^/^+^ cells. A; PDTC represses SOD1 activity in U937 cells. B; SOD1 activity is not sensitive to PDTC in JNK deficient cells. Serum starved U937, JNK ^-^ **/** ^**-**^ and JNK ^**+**^ **/** ^**+**^ cells were treated with 1 μM PDTC in RPMI1640 media supplemented with 1% serum. After 24 h of treatment, cells were lysed and SOD1 activity in cell lysates was assayed as described in material & methods. The results are presented as mean ± SE from 4 separate experiments. *P < 0.01 vs. control. NS, not significant.

To determine the functional significance of JNK pathway in modulating SOD1 gene, we evaluated the effects of PDTC on SOD1 enzymatic activity in U937 cells. As shown in Fig 8A, PDTC treatment reduced significantly SOD1 activity. To confirm further that JNK pathway is required for the full action of PDTC on SOD1 activity, we used JNK deficient cells to evaluate SOD1 activity in the presence or absence of PDTC. Consistent with the results obtained with U937 cells, activity of SOD1 was markedly decreased in JNK^+/+^ mouse fibroblast cell line in the presence of PDTC (Fig 8B). In contrast, using JNK deficient cells the PDTC action was lost in the absence of JNK pathway (Fig 8B). As reported for SOD1 promoter activity (Fig 6), SOD1 basal enzymatic activity in JNK deficient cells was 3 fold higher than in wild type cells (Fig 8B). Taken together, these findings demonstrate that JNK pathway plays an important role in regulating SOD1 transcription and enzymatic activity.

### JNK deficient cells do not produce ROS in the presence of PDTC

**Fig 9:**
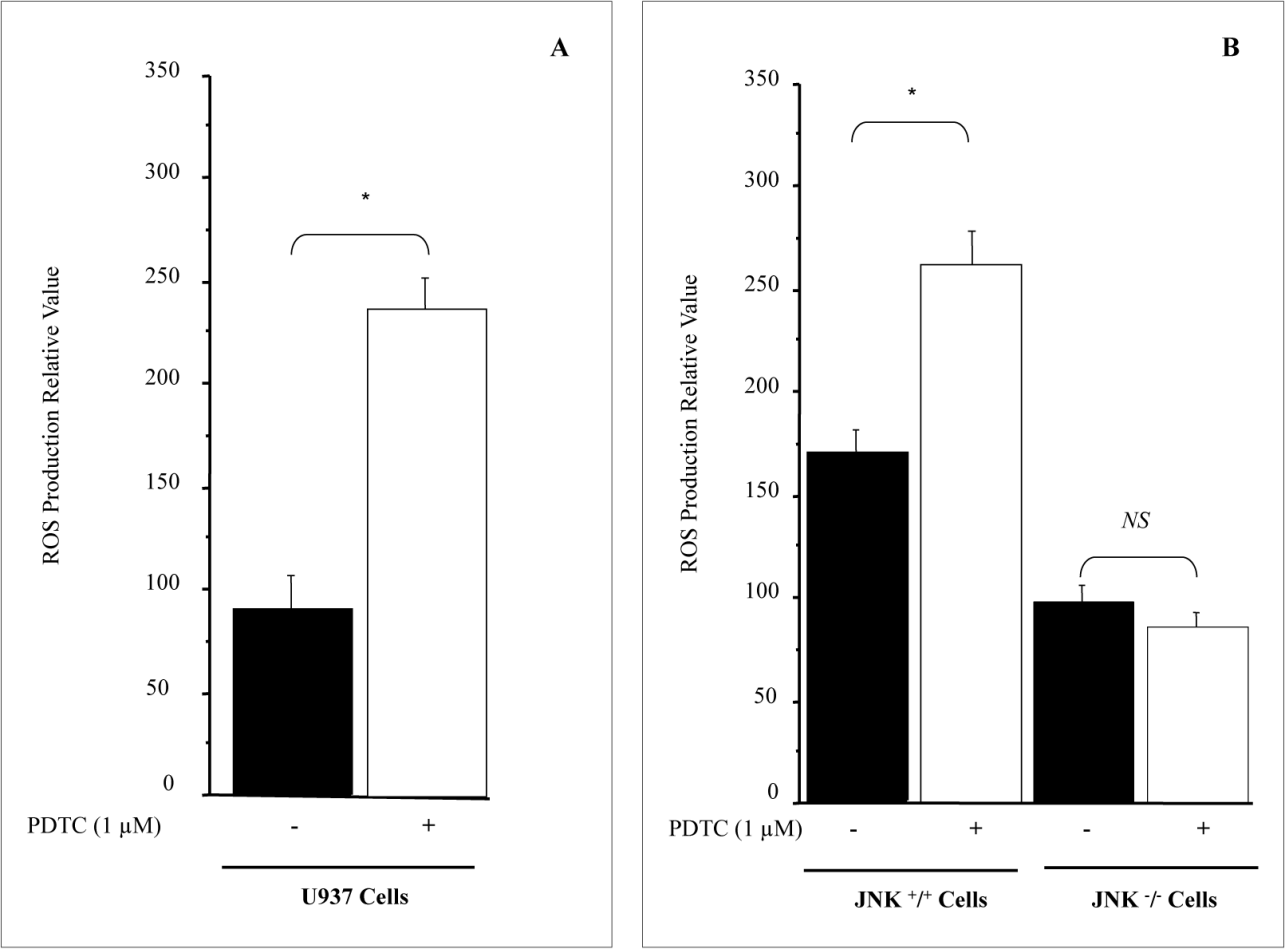
JNK deficient cells do not produce ROS in the presence of PDTC. ROS production under PDTC treatment requires active JNK pathway. A; PDTC induces ROS production in U937 cells. B; Serum starved JNK ^-^ **/** ^**-**^ and JNK ^**+**^ **/** ^**+**^ cells were treated with PDTC in RPMI 1640 media supplemented with 1% serum. After 1 h of treatment, cells were lysed and ROS levels were determined as described in material & methods. The results are presented as mean ± SE from 4 separate experiments. *P < 0.01 vs. control. NS, not significant.

To determine the functional significance of JNK in modulating SOD1 enzymatic activity, we measured ROS production in U937 cells using the probe 2, 7-dichlorofluorescein (DCF). We found that the ROS level was markedly increased in cell treated with PDTC (Fig 9A). To confirm further that JNK pathway is required for the full action of PDTC on SOD1 activity, we used JNK deficient cells to evaluate ROS production. Consistent with the results obtained with U937 cells, ROS production was increased in JNK^+/+^ mouse fibroblast cell line in the presence of PDTC (Fig 9B). In contrast, using JNK deficient cells the PDTC action was lost in the absence of JNK pathway (Fig 9B). Taken together, these findings suggest that PDTC plays an important role in regulating ROS levels, at least in part, by modulating intracellular SOD1 activity.

## Discussion

Oxidative stress, a cellular imbalance between production and elimination of reactive oxygen species (ROS), causes ROS accumulation and plays an important role in tumor biology as essential intracellular messengers involved in cancer progression. Recent clinical and experimental findings suggest that oxidative stress is associated with the progression of breast cancer [36–39]. PDTC suppresses tumor angiogenesis, growth and migration of breast cancer via inhibiting paracrine and autocrine effects of VEGF through the reduction of NF-κB activation and VEGF expression. Intravenous administration of PDTC reduced tumor growth in nude mice using MCF-7 sphere cells as model of breast cancan stem-like cells [40].

Although PDTC is an inhibitor of NF-κB activation in various cell types that ultimately results in the inhibition of apoptosis [16], PDTC is also a pro-oxidant capable of inducing cell death in PC12 cells [24]. In order to understand the relation between NF-κB inactivation and PDTC pro-oxidant effects, we examined whether SOD1 could be one of the target gene of PDTC treatment in hematopoietic human cancer cell line U937.

An important finding of this study is that PDTC down-regulated SOD1 transcripts and protein in dose-dependent manner. SOD1 inhibition may result in a decrease in the protective effect of SOD1 against oxidative stress and cell damage. In fact previous studies suggested that SOD1 might be of critical importance for the maintenance of the integrity of the organelle in breast cancer cells [41]. Overexpression of SOD1 in cancer cells may act as adaptation mechanism to maintain total ROS levels below a critical threshold so that the integrity of the organelle is maintained. In agreement with this suggestion, we observed that inhibition of SOD1 enzymatic activity by PDTC was associated with a marked accumulation of ROS in cells. These observations my explained the mechanism of inhibition of tumor angiogenesis and growth of breast cancer in female mice induced by ROS accumulation after oral administration of PDTC [23].

Much has been learned over the past years concerning the molecular events involved in the transcriptional regulation of genes by antioxidants, but no report has investigated the mechanisms involved in gene repression by PDTC. As reported earlier [30], inhibition of the NF-κB pathway by pretreatment of U937 cells with PDTC, N-acetylcysteine (NAC), or sulfasalazine did not prevent SOD1 down-regulation by TNF-α, but does inhibit basal SOD1 transcription, suggesting that NF-κB acts as an SOD1 promoter co-activator as recently reported [42]. NF-κB is master coordinator of inflammatory and immune responses [43, 44], and also promotes cell survival [45–47].

Assessing SOD1 promoter activity also delineated the molecular processes involved in PDTC regulation of SOD1 expression. The human SOD1 promoter activity is dependent on the Sp1, Egr-1, and C/EBP family of transcription factors [30]. Using the same constructs, deletion experiments indicated that the PDTC-response element is located downstream -157 bp of SOD1 5′-flanking DNA. Within this region, we identified several motifs that are homologous to known cis- acting regulatory elements including Sp1, Egr-1, and C/EBP (AP-1 inverted half site), as similarly described [33]. In contrast, no NF-κB binding site was found in this region as previously reported [30], suggesting that PDTC causes posttranslational changes, probably through c-Jun phosphorylation and AP-1/NF-κB negative interaction. Since AP-1 was found to be a key regulator of SOD1 repression by TNF-α [30], the involvement of AP-1 in SOD1 gene regulation by PDTC was examined. AP-1 is known to act through protein/protein or protein/DNA interaction. AP-1 is likely involved in the PDTC regulation of the SOD1 gene based on several observations. First, PDTC induces both AP-1-binding and promoter activity in our system. Second, AP-1 inactivation by pre-incubation of cells with specific JNK inhibitor blocked SOD1 promoter repression by PDTC. Third, overexpression of NF-κB p65 subunit in cells transfected with SOD1 promoter didn’t activate the promoter activity. In contrast, in the presence of JNK inhibitor, ectopic NF-κB p65 subunit induces SOD1 promoter activity. Furthermore, the absence of transcription activation by p65 overexpression suggests that JNK interacts negatively with NF-κB.

In this study, we also investigated the signaling pathways involved in PDTC effects on SOD1 gene. As PDTC is known to activate MAP kinase JNK, this pathway was considered. Our data showed that PDTC caused an activation of JNK in U937 cells, suggesting that JNK signaling pathway may be involved in PDTC modulation of SOD1 expression.

Work from different laboratories has demonstrated the possibility of a cross talk between the JNK and NF-κB pathway [47–49]. To address this point, we used mouse cells deficient in JNK1/JNK2. We have found that deletion of JNK modifies the response of the SOD1 gene to PDTC.

The JNK inhibitor and JNK deficient cells indicated that JNK activation was required for the repression of SOD1 expression by PDTC. Additionally, our studies showed that AP-1 was the transcription factor involved in SOD1 repression. Our data indicated that JNK activation and c-Jun phosphorylation lead to AP-1 activation (a direct substrate of JNK) and that AP-1 activation was required for the down regulation of SOD1 by PDTC. Our results are in line with other studies reporting that JNK inhibits NF-κB. Such cross talk between the mediators has been noted previously in fibroblasts, where JNK was found to inhibit NF-κB [50].

The regulation of c-Jun activation by PDTC takes place through activation of JNK that phosphorylates c-Jun and increases its transactivation potential. Activation of JNK occurs as a consequence of activation of MEKK1 and the downstream kinase MEKK4/SEK1, the final JNK activator [51, 52].

Finally, the data presented here support the hypothesis that SOD1 is a potential therapeutic target for the treatment of cancer and that inhibiting SOD1 results in the down-regulation of multiple signaling pathways important for tumor cell function [53]. It can be speculated that the anti-tumor growth of PDTC observed in vivo in breast cancer mice model my involve SOD1 repression through JNK/AP-1 signaling pathway, leading to ROS accumulation in tumors. Furthermore, elucidating the pro-oxidant cell death mechanism of PDTC in various cancers allows for a potential treatment either as single agent or in combination with other known cancer therapeutics.

## References

[1] Jacobson MD. Reactive oxygen species and programmed cell death. Trends Biochem. Sci. 1996; 21:83–86. 8882579

[2] Fuchs D, Baier-Bitterlich G, Wede I, Wachter H. Oxidative Stress and the Molecular Biology of Antioxidant Defenses. Scandalios JG (ed.). CSHL Press: Cold Spring Harbor, NY; 1997 Pp. 139–168.

[3] Rosen DR, Siddique T, Patterson D, Figlewicz DA, Sapp P, Hentati A, et al Mutations in Cu/Zn superoxide dismutase gene are associated with familial amyotrophic lateral sclerosis. Nature 1993; 362: 59–62. 8446170 10.1038/362059a0

[4] Kong J, Xu Z. Massive mitochondrial degeneration in motor neurons triggers the onset of amyotrophic lateral sclerosis in mice expressing a mutant SOD1. J Neurosci. 1998; 18: 3241–3250. 9547233 10.1523/JNEUROSCI.18-09-03241.1998 PMC6792665

[5] Wheeler MD, Thurman RG. Up-regulation of CD14 in liver caused by acute ethanol involves oxidant-dependent AP-1 pathway. J Biol Chem. 2003; 278: 8435–8441. 12482856 10.1074/jbc.M212076200

[6] Lin SJ, Shyue SK, Hung YY, Chen YH, Ku HH, Chen JW, et al Superoxide dismutase inhibits the expression of vascular cell adhesion molecule-1 and intracellular cell adhesion molecule-1 induced by tumor necrosis factor-alpha in human endothelial cells through the JNK/p38 pathways. Arterioscler Thromb Vasc Biol. 2005; 25: 334–340. 15576639 10.1161/01.ATV.0000152114.00114.d8

[7] Chen YR, Tan TH. The c-Jun N-terminal kinase pathway and apoptotic signaling. Int J Oncol. 2000; 16: 651–662. 10717232 10.3892/ijo.16.4.651

[8] Ip YT, Davis RJ. Signal transduction by the c-Jun N-terminal kinase (JNK)-from inflammation to development. Curr Opin Cell Biol. 1998; 10: 205–219. 9561845 10.1016/s0955-0674(98)80143-9

[9] Schaeffer HJ, Weber MJ. Mitogen-activated protein kinases: specific messages from ubiquitous messengers. Mol Cell Biol. 1999; 19: 2435–2444. 10082509 10.1128/mcb.19.4.2435 PMC84036

[10] Chen M, Hu DN, Pan Z, Lu CW, Xue CY, Aass I. Curcumin protects against hyperosmoticity-induced IL-1beta elevation in human corneal epithelial cell via MAPK pathways. Exp Eye Res. 2010; 90: 437–443. doi: 10.1016/j.exer.2009.12.004 20026325 10.1016/j.exer.2009.12.004

[11] Lehmann TG, Wheeler MD, Froh M, Schwabe RF, Bunzendahl H, Samulski RJ, et al Effects of three superoxide dismutase genes delivered with an adenovirus on graft function after transplantation of fatty livers in the rat. Transplantation 2003; 76: 28–37. 12865782 10.1097/01.TP.0000065299.29900.17

[12] Wheeler MD, Katuna M, Smutney OM, Froh M, Dikalova A, Mason RP, et al Comparison of the effect of adenoviral delivery of three superoxide dismutase genes against hepatic ischemia-reperfusion injury. Hum Gene Ther. 2001; 12: 2167–2177. 11779401 10.1089/10430340152710513

[13] Wheeler MD, Kono H, Yin M, Rusyn I, Froh M, Connor HD, et al Delivery of the Cu/Zn-superoxide dismutase gene with adenovirus reduces early alcohol-induced liver injury in rats. Gastroenterology 2001; 120: 1241–1250. 11266387 10.1053/gast.2001.23253

[14] Verhaegen S, McGowan AJ, Brophy AR, Fernandes RS, Cotter TG. Inhibition of apoptosis by antioxidants in the human HL-60 leukemia cell line. Biochem Pharmacol. 1995; 50: 1021–1029. 7575657 10.1016/0006-2952(95)00233-p

[15] Chinery R, Brockman JA, Peeler MO, Shyr Y, Beauchamp RD, Coffey RJ. Antioxidants enhance the cytotoxicity of chemotherapeutic agents in colorectal cancer: a p53-independent induction of p21WAF1/CIP1 via C/EBPβ. Nat Med. 1997; 3: 1233–1241. 9359698 10.1038/nm1197-1233

[16] Nobel CI, Kimland M, Lind B, Orrenius S, Slater AF. Dithiocarbamates induce apoptosis in thymocytes by raising the intracellular level of redox-active copper. J Biol Chem. 1995; 270: 26202–26208. 7592825 10.1074/jbc.270.44.26202

[17] Burkitt MJ, Bishop HS, Milne L, Tsang SY, Provan GJ, Nobel CS, et al Dithiocarbamate toxicity toward thymocytes involves their copper-catalyzed conversion to thiuram disulfides, which oxidize glutathione in a redox cycle without the release of reactive oxygen species. Arch Biochem Biophys. 1998; 353: 73–84. 9578602 10.1006/abbi.1998.0618

[18] Taylor KL, Ziegler DM. Studies on substrate specificity of the hog liver flavin-containing monooxygenase. Anionic organic sulfur compounds. Biochem Pharmacol. 1987; 36: 141–146. 3801050 10.1016/0006-2952(87)90391-1

[19] Nemeth ZH, Hasko G, Vizi ES. Pyrrolidine dithiocarbamate augments IL-10, inhibits TNF-α, MIP-1α, IL-12, and nitric oxide production and protects from the lethal effect of endotoxin. Shock 1998; 10: 49–53. 9688091 10.1097/00024382-199807000-00009

[20] Liu SF, Ye X, Malik AB. Inhibition of NF-κB activation by pyrrolidine dithiocarbamate prevents in vivo expression of pro-inflammatory genes. Circulation 1999; 100: 1330–1337. 10491379 10.1161/01.cir.100.12.1330

[21] Ziegler-Heitbrock HW, Sternsdorf T, Liese J, Belohradsky B, Weber C, Wedel A, et al Pyrrolidine dithiocarbamate inhibits NF-κB mobilization and TNF production in human monocytes. J Immunol. 1993; 151: 6986–6993. 8258705

[22] Munoz C, Pascual-Salcedo D, Castellanos MC, Alfranca A, Aragones J, Vara A, et al Pyrrolidine dithiocarbamate inhibits the production of interleukin-6, interleukin-8, and granulocyte-macrophage colony-stimulating factor by human endothelial cells in response to inflammatory mediators: modulation of NF-κB and AP-1 transcription factors activity. Blood 1996; 88: 3482–3490. 8896414

[23] Gu JW, Young E, Busby B, Covington J, Tan W, Johnson JW. Oral administration of pyrrolidine dithiocarbamate (PDTC) inhibits VEGF expression, tumor angiogenesis and growth of breast cancer in female mice. Cancer Biology & Therapy 2009; 8: 514–521. 19242105 10.4161/cbt.8.6.7689

[24] Chung KC, Park JH, Kim CH, Lee HW, Sato N, Uchiyama Y, et al Novel biphasic effect of pyrrolidine dithiocarbamate on neuronal cell viability is mediated by the differential regulation of intracellular zinc and copper ion levels, NF-κB, and MAP kinases. J Neurosci Res. 2000; 59: 117–125. 10658192

[25] Meisner M, Schmidt J, Schywalsky M, Tschaikowsky K. Influence of pyrrolidine dithiocarbamate on the inflammatory response in macrophages and mouse endotoxin shock. Int J Immunopharmacol. 2000; 22: 83–90. 10684991 10.1016/s0192-0561(99)00071-5

[26] Herrmann JL, Beham AW, Sarkiss M, Chiao PJ, Rands MT, Bruckheimer EM, et al Bcl-2 suppresses apoptosis resulting from disruption of the NF-κB survival pathway. Exp Cell Res. 1997; 237: 101–109. 9417872 10.1006/excr.1997.3737

[27] Matroule JY, Carthy CM, Granville DJ, Jolois O, Hunt DW, Piette J. Mechanism of colon cancer cell apoptosis mediated by pyropheophorbide-a methylester photosensitization. Oncogene 2001; 20: 4070–4084. 11494135 10.1038/sj.onc.1204546

[28] Malaguarnera L, Pilastro MR, DiMarco R, Scifo C, Renis M, Mazzarino MC, et al Cell death in human acute myelogenous leukemic cells induced by pyrrolidine dithiocarbamate. Apoptosis 2003; 8: 539–545. 14601560 10.1023/a:1025550726803

[29] Di Nicuolo F, Serini S, Boninsegna A, Palozza P, Calviello G. Redox regulation of cell proliferation by pyrrolidine dithiocarbamate in murine thymoma cells transplanted in vivo. Free Radical Biol Med. 2001; 31: 1424–1431. 11728814 10.1016/s0891-5849(01)00714-6

[30] Afonso V, Santos G, Collin P, Khatib AM, Mitrovic DR, Lomri N, et al Tumor necrosis factor-alpha down-regulates human Cu/Zn superoxide dismutase 1 promoter via JNK/AP-1 signaling pathway. Free Radic Biol Med. 2006; 41: 709–721. 16895791 10.1016/j.freeradbiomed.2006.05.014

[31] Sabapathy K, Jochum W, Hochedlinger K, Chang L, Karin M, Wagner EF. Defective neural tube morphogenesis and altered apoptosis in the absence of both JNK1 and JNK2. Mech Dev. 1999; 89: 115–124. 10559486 10.1016/s0925-4773(99)00213-0

[32] Verrecchia F, Tacheau C, Wagner EF, Mauviel A. A central role for the JNK pathway in mediating the antagonistic activity of pro-inflammatory cytokines against transforming growth factor-beta-driven SMAD3/4-specific gene expression. J Biol Chem. 2003; 278:1585–1593. 12426318 10.1074/jbc.M206927200

[33] Minc E, de Coppet P, Massoni P, Thiery L, Dutertre S, Amor-Guéret M, et al The human copper-zinc superoxide dismutase gene (SOD1) proximal promoter is regulated by Sp1, Egr-1, and WT1 via non-canonical binding sites. J Biol Chem. 1999; 274: 503–509. 9867871 10.1074/jbc.274.1.503

[34] Tzagarakis-Foster C, Geleziunas R, Lomri A, An J, Leitman DC. Estradiol represses human T-cell leukemia virus type 1 Tax activation of tumor necrosis factor-alpha gene transcription. J Biol Chem. 2002; 277: 44772–44777. 12237295 10.1074/jbc.M205355200

[35] Dignam JD, Lebovitz RM, Roeder RG. Accurate transcription initiation by RNA polymerase II in a soluble extract from isolated mammalian nuclei. Nucleic Acids Res. 1983; 11: 1475–1489. 6828386 10.1093/nar/11.5.1475 PMC325809

[36] Rossner P Jr, Gammon MD, Terry MB, Agrawal M, Zhang FF, Teitelbaum SL, et al Relationship between urinary 15-F2t-Isoprostane and 8-oxodeoxyguanosine levels and breast cancer risk. Cancer Epidemiol Biomarkers Prev. 2006; 15: 639–644. 16614103 10.1158/1055-9965.EPI-05-0554

[37] Portakal O, Ozkaya O, Inal ME, Bozan B, Kosan M, Sayek I. Coenzyme Q10 concentrations and antioxidant status in tissues of breast cancer patients. Clin Biochem. 2000; 33: 279–284. 10936586 10.1016/s0009-9120(00)00067-9

[38] Ambrosone CB. Oxidants and antioxidants in breast cancer. Antioxid Redox Signal 2000; 2: 903–917. 11213491 10.1089/ars.2000.2.4-903

[39] Karihtala P, Winqvit R, Syvaoja JE, Kinnula VL, Soini Y. Increasing oxidative damage and loss of mismatch repair enzymes during breast carcinogenesis. Eur J Cancer 2006; 42: 2653–2659. 16996262 10.1016/j.ejca.2006.05.037

[40] Zhou J, Zhang H, Gu P, Bai J, Margolick JB, Zhang Y. NF-κB pathway inhibitors preferentially inhibit breast cancer stem-like cells. Breast Cancer Res Treat. 2008; 111: 419–427. 17965935 10.1007/s10549-007-9798-y PMC3320112

[41] Papa L, Hahn M, Marsh EL, Evans BS, Germain D. SOD2 to SOD1 switch in breast cancer. J Biol Chem. 2014; 289: 5412–5416. doi: 10.1074/jbc.C113.526475 24448804 10.1074/jbc.C113.526475 PMC3937618

[42] Rojo AI, Salinas M, Martín D, Perona R, Cuadrado A. Regulation of Cu/Zn-superoxide dismutase expression via the phosphatidylinositol 3 kinase/Akt pathway and nuclear factor-κB. J Neurosci. 2004; 24: 7324–7334. 15317858 10.1523/JNEUROSCI.2111-04.2004 PMC6729771

[43] Chen LF, Greene WC. Shaping the nuclear action of NF-κB. Nat Rev Mol Cell Biol. 2004; 5: 392–401. 15122352 10.1038/nrm1368

[44] Li Q, Verma IM. NF-κB regulation in the immune system. Nat Rev Immunol. 2002; 2: 725–734. 12360211 10.1038/nri910

[45] Kucharczak J, Simmons MJ, Fan Y, Gélinas C. To be, or not to be: NF-κB is the answer role of Rel/NF-κB in the regulation of apoptosis. Oncogene 2003; 22: 8961–8982. 14663476 10.1038/sj.onc.1207230

[46] Orlowski RZ, Baldwin AS. NF-κB as a therapeutic target in cancer. Trends Mol Med. 2002; 8: 385–389. 12127724 10.1016/s1471-4914(02)02375-4

[47] Maggirwar SB, Ramirez S, Tong N, Gelbard HA, Dewhurst S. Functional interplay between nuclear factor-κB, and c-Jun integrated by coactivator p300 determines the survival of nerve growth factor-dependent PC12 cell. J Neurochem. 2000; 74: 527–539. 10646503 10.1046/j.1471-4159.2000.740527.x

[48] Smaele E, Zazzeroni P, Nguyen DU, Jin R, Jones J, Cong R, et al Induction of gadd45b by NF-κB downregulates pro-apoptotic JNK signaling. Nature 2001; 414: 308–313. 11713530 10.1038/35104560

[49] Tang G, Minemoto Y, Dibling B, Purcell NH, Li Z, Karin M, et al Inhibition of JNK activation through NF-κB target genes. Nature 2001; 414: 313–317. 11713531 10.1038/35104568

[50] Sánchez-Pérez I, Benitah SA, Martínez-Gomariz M, Lacal JC, Perona R. Cell stress and MEKK1-mediated c-Jun activation modulate NF-κB activity and cell viability. Mol Biol Cell. 2002; 13: 2933–2945. 12181357 10.1091/mbc.E02-01-0022 PMC117953

[51] Sánchez-Perez I, Murguía JR, Perona R. Cisplatin induces a persistent activation of JNK that is related to cell death. Oncogene 1998; 16: 533–540. 9484843 10.1038/sj.onc.1201578

[52] Chen YR, Wang X, Templeton D, Davis RJ, Tan TH. The role of c-Jun N-terminal kinase (JNK) in apoptosis induced by ultraviolet C and gamma-radiation. J Biol Chem. 1996; 271: 31929–31936. 8943238 10.1074/jbc.271.50.31929

[53] Glasauer A, Sena LA, Diebold LP, Mazar AP, Chandel NS. Targeting SOD1 reduces experimental non-small-cell lung cancer. J Clin Invest. 2013; 124: 117–128. 10.1172/JCI71714 PMC3871252 24292713

